# Mitochondrial Dysfunction and Oxidative Stress in Asthma: Implications for Mitochondria-Targeted Antioxidant Therapeutics

**DOI:** 10.3390/ph4030429

**Published:** 2011-02-25

**Authors:** P. Hemachandra Reddy

**Affiliations:** 1 Neurogenetics Laboratory, Division of Neuroscience, Oregon National Primate Research Center, Oregon Health and Science University, 505 NW 185th Avenue, Beaverton, OR 97006, USA; 2 Department of Physiology and Pharmacology, Oregon Health and Science University, Portland, OR 97201, USA; E-Mail: reddyh@ohsu.edu; Tel.: 503-418-2625; Fax: 503-418-2701

**Keywords:** asthma, mitochondrial dysfunction, mitochondria-targeted antioxidants, reactive oxygen species

## Abstract

Asthma is a complex, inflammatory disorder characterized by airflow obstruction of variable degrees, bronchial hyper-responsiveness, and airway inflammation. Asthma is caused by environmental factors and a combination of genetic and environmental stimuli. Genetic studies have revealed that multiple loci are involved in the etiology of asthma. Recent cellular, molecular, and animal-model studies have revealed several cellular events that are involved in the progression of asthma, including: increased Th2 cytokines leading to the recruitment of inflammatory cells to the airway, and an increase in the production of reactive oxygen species and mitochondrial dysfunction in the activated inflammatory cells, leading to tissue injury in the bronchial epithelium. Further, aging and animal model studies have revealed that mitochondrial dysfunction and oxidative stress are involved and play a large role in asthma. Recent studies using experimental allergic asthmatic mouse models and peripheral cells and tissues from asthmatic humans have revealed antioxidants as promising treatments for people with asthma. This article summarizes the latest research findings on the involvement of inflammatory changes, and mitochondrial dysfunction/oxidative stress in the development and progression of asthma. This article also addresses the relationship between aging and age-related immunity in triggering asthma, the antioxidant therapeutic strategies in treating people with asthma.

## 1. Introduction

Asthma is a complex inflammatory disorder characterized by airflow obstruction of variable degrees, bronchial hyper-responsiveness, and airway inflammation [1]. Asthma affects people of all ages, but children are mostly affected. Currently in the United States, some 22 million people are reported to have asthma, 6 million of whom are children. Asthma is a heterogeneous disorder caused by environmental factors and a combination of genetic and environmental stimuli. Recent genetic studies have revealed that multiple genetic loci are involved in causing the etiology of asthma [2-5]. However, the ADAM33 gene (located on chromosome 20), expressed predominantly in lung and muscle cells, is strongly associated with the disorder [6]. In addition, there is some evidence indicating the possible involvement of mitochondrial DNA (mtDNA) defects in the etiology of asthma [7-9].

Recent molecular, cellular, and animal model studies have revealed that several cellular events are involved in the progression of asthma and in the subsequent injury to the tissues of the bronchial epithelium, including increased IgE synthesis, an imbalanced T helper-type 1/T helper-type 2 (Th1/Th2) paradigm, increased Th2 cytokines leading to the recruitment of inflammatory cells to the airway and the activation of those cells; increased production of reactive oxygen species (ROS); and mitochondrial damage and dysfunction in activated inflammatory cells [10,11].

This article discusses the association of genes with asthma, and mitochondrial dysfunction and oxidative stress in asthma, particularly in asthmatic mouse models and in peripheral tissues from asthmatic humans. This article also discusses the potential antioxidant therapeutics of asthma, with a focus on mitochondria-targeted antioxidants.

## 2. Causes of Asthma

Asthma is a complex disorder of the airways, in which antigens trigger Th2 pulmonary inflammation, resulting in the infiltration of eosinophils into the lungs, airway hyper-reactivity, mucus hypersecretion, and increased production of IgE [1,12,13]. In asthmatic patients, the muscles of airway walls squeeze, the walls of the airway swell, and the airway narrows, blocking the passage of air. Mucus produced inside the lining of the airway further blocks air passage. Asthma is a major, world-wide health concern; it affects 7% of the U.S. population and 300 million worldwide [14]. Asthma is believed to be involved in interactions between genes and environmental factors. Once a person develops asthma, many environmental factors (natural and man-made) may trigger an asthma attack [15]. Such factors include animal skin; hair and feathers; grains; dust and house mites; exercise; mold; pollen; smoke; strong odors and chemical sprays; occupational dust, such as from wood and metals; and air pollution, such as cigarette smoke, auto exhaust, and sulfur dioxide [16-18].

## 3. Genetics and Asthma

Recent genetic studies of asthmatic humans have revealed that multiple genetic loci are involved in triggering asthma [2-5]. Over 25 genetic loci have been found to be associated with asthma in 6 or more separate ethnic populations [19], and many of these genes are associated with the immune system. The ADAM33 gene (located on chromosome [20]), expressed mainly in lung and muscle cells, has been particularly associated with asthma [6]. Gene and environmental interactions may cause a vast majority of asthma cases [15]. However, a recent report suggests that childhood asthma may be associated with single, nucleotide polymorphisms in genetic loci on chromosome 17 [20].

A study of a well-known childhood asthma management program revealed that single nucleotide polymorphisms (SNPs) may be associated with asthma; these SNPs are found in multiple genetic loci, including importin 13 [21], VEGF [22], MMP-12 [23], Wnt [24], ORMDL3, 2PBP2/GSDMB/ORMDL3 [25-27], PDE4D [28], PRKCA [29], TGF-beta1 [30], IL-10 [31], IL-13 [32], JAG1 [33], ANKRD5 [33], IL-17 [34], IL25 [35], 12q24 [36-39], IL-12beta [40], and beta2-adrenergic receptor [41]. In addition, mtDNA defects may be involved in the progression of asthma in children [7-9].

### 3.1. Mitochondrial DNA Changes and Asthma

Polymorphisms or haplotype differences in the mitochondrial genome are associated with asthma in humans [7-9]. Raby *et al.* [9] investigated the involvement of variations in the mitochondrial genome, in children with asthma and atopy. They studied 654 self-reporting white children (5 to 12 years old) who had mild to moderate asthma. Eight haplogroup-tagging polymorphisms were genotyped with TaqMan probe hybridization assays in this population, and mitochondrial haplogroup tests of association with atopy-related phenotypes were performed with haplo-stats. Raby *et al*. found strong evidence associating the mitochondrial haplogroup with total serum IgE levels (684 IU/L in carriers and 389 IU/L in non-carriers) and with carriers of European haplogroup U (frequency 11%) who have higher total serum IgE levels compared to noncarriers. Haplogroup U carriers reported greater reactivity to skin pricks and a higher frequency of atopic dermatitis. This study suggested that common mitochondrial haplogroups may influence atopic diathesis, particularly in children with a maternal history of atopy.

**Figure 1:**
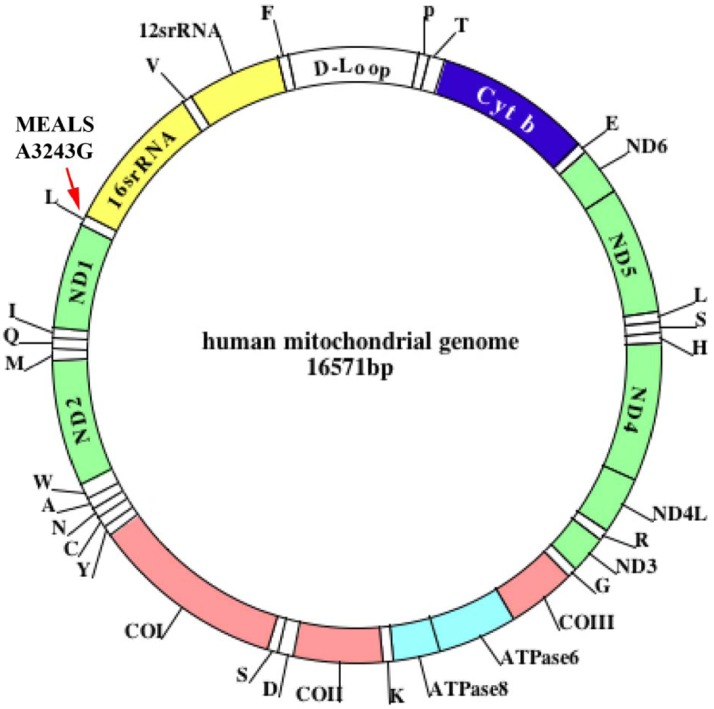
The structure of human mtDNA. The mitochondrial genome is circular and has 2 strands: a guanine-rich outer strand and cytosine-rich inner strand. mtDNA is encoded with 13 polypeptide chains, all of which are essential components of the electron transport chain. mtDNA also encodes 22 tRNAs (individual capital letters of outer strand represent each tRNA in the figure), 12S, and 16S rRNA. The D-loop represents the control region of mtDNA. The somatic mtDNA mutations have been reported to be elevated in PD, AD and HD. Mitochondrial haplotype U has been associated with asthma, and mitochondrial MELAS A3243G (represented with red arrow in the figure) has been associated with a rare form of asthma.

Jones *et al*. [8] sought to determine whether mtDNA mutations play a role in the development of age-related maculopathy. Using PCR and restriction fragment length polymorphism analysis they detected mitochondrial myopathy, encephalopathy, lactic acidosis, and the stroke (MELAS) A3243G mutation in 570 subjects identified to have signs of early, age-related maculopathy. In one patient, they found an A3243G mutation. This patient exhibited clinical symptoms of early, age-related maculopathy, mild hearing loss, hypertension, and ischemic heart disease, indicating that the mitochondrial A3243G mutation is involved in a rare form of asthma (Figure 1).

### 3.2. Mitochondrial DNA Changes and Aging

Human mtDNA defects have been found to accumulate with age and in age-related neurodegenerative diseases, such as Alzheimer's and Parkinson's [42-46] (Figure 1). These defects are known to induce free-radical production and to damage mitochondrial function [47-48]. In turn, oxidative stress-related free radicals further damage mtDNA, leading to large deletions and point mutations in various tissues of rodents and humans [49-54]. Large mtDNA deletions have consistently been reported to increase with age in humans [49-54]. In studies of mtDNA, Jessie and colleagues [55] found high numbers of mtDNA deletions in tissue from prostate cancer patients compared to the number of mtDNA deletions in non-cancerous tissues from these same patients, suggesting that mtDNA may be related to prostate cancer [54,55]. They also found high numbers of mtDNA deletions in patients with end-stage renal disease [55]. mtDNA defects, including large deletions and point mutations, may be present in the lungs of elderly people with asthma.

These age-dependent mtDNA defects may be critical factors determining the susceptibility of asthma in elderly people. However, further research is needed to investigate mtDNA defects, including large deletions and single nucleotide changes in the lungs of asthmatic aged people and children.

## 4. Mitochondrial Structure, Function, and Physiology

Increasing evidence suggests that abnormalities in mitochondria are involved not only in aging, age-related neurodegenerative diseases, cancer, diabetes, and several other mitochondrial diseases [45,47,48,56-58], but also in the development of asthma [7-9]. However, the precise connection between mitochondrial abnormalities and asthma is not well understood.

**Figure 2:**
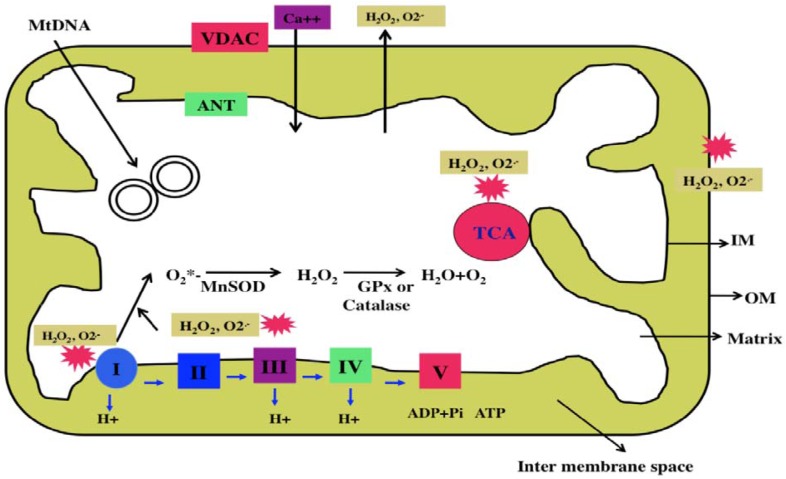
Structure of mitochondrion. A mitochondrion is compartmentalized with 2 lipid membranes: the inner mitochondrial membrane and the outer mitochondrial membrane. The inner mitochondrial membrane houses the mitochondrial respiratory chain and provides a highly efficient barrier to ionic flow. In the electron transport chain, complexes I and III leak electrons to oxygen, producing primarily superoxide radicals. Superoxide radicals are dismutated by manganese superoxide dismutase and produce H_2_O_2_. In addition, the electron transport chain involves H_2_O_2_ reducing to H_2_O and O_2_ by catalase or by glutathione peroxidase accepting electrons donated by NADH and FADH_2_ and then yielding energy from which ATP is generated from adenosine diphosphate and inorganic phosphate. Free radicals are also generated by tricarboxylic acid in the matrix.

Mitochondria are cytoplasmic organelles that are essential for life and death. Mitochondria perform several cellular functions, including intracellular calcium regulation, ATP production, the release of proteins that activate the caspase family of proteases, alteration of the reduction-oxidation potential, and free-radical scavenging [65]. Mitochondria are compartmentalized into 2 lipid membranes: the outer mitochondrial membrane and the inner mitochondrial membrane. The outer membrane is porous and allows the passage of low molecular-weight substances between the cytosol and the inter-membrane space. The inner membrane provides a highly efficient barrier to ionic flow, houses the mitochondrial respiratory chain (*i.e.*, the electron transport chain [ETC]), and covers the mitochondrial matrix, which contains tricarboxylic acid (TCA) and beta-oxidation (Figure 2). Mitochondria are transmitted maternally. However, in rare situations, paternal inheritance and a recombination of mtDNA have been reported [60].

Mitochondria are controlled by both nuclear and mitochondrial genomes. mtDNA consists of a 16,571 base-pair, double-stranded, circular DNA molecule [61]. A mitochondrion contains 2–10 copies of mtDNA [60]. mtDNA contains 13 polypeptide genes that encode essential components of the ETC. mtDNA also encodes the 12S and 16S rRNA genes, and the 22 tRNA genes required for mitochondrial protein synthesis [60]. Nuclear genes encode the remaining mitochondrial proteins, metabolic enzymes, DNA and RNA polymerases, ribosomal proteins, and mtDNA regulatory factors, such as the mitochondrial transcription factor A. Nuclear mitochondrial proteins are synthesized in the cytoplasm and are subsequently transported into mitochondria. Mitochondrial ATP is generated via oxidative phosphorylation within the inner mitochondrial membrane (Figure 2).

Oxidative stress is a major factor associated with the development and progression of asthma. A large body of data suggests that free radical oxidative damage—particularly of lipids, nucleic acids, and proteins—is extensive in the lungs of ovalbumin (OVA)-sensitized asthmatic mice [10,11,62,63]. Increased oxidative stress is thought to result in the generation of ROS, which is released by activated inflammatory cells in the epithelium.

Oxidative stress is commonly used to explain the imbalance between the production of oxidants and endogenous antioxidant defenses in mammalian cells. In general, mammalian cells undergo apoptotic death when there is an imbalance between oxidants and antioxidants (that is, the cell has more oxidants than endogenous antioxidant defenses). This oxidative damage mainly occurs via the mitochondrial ETC [64,65].

The production of mitochondrial superoxide radicals (O_2_*^−^) occurs primarily at discrete points in the ETC, at complexes 1 and 3, and in TCA components, including α-ketoglutarate dehydrogenase [64,65]. In addition, mitochondrial O_2_*^−^ are generated in the outer mitochondrial membrane. Flavoprotein (monoamine oxidase), localized on the outer mitochondrial membrane, catalyzes the oxidative deamination of primary aromatic amines. This deamination is a quantitatively large source of superoxide radicals and hydrogen peroxide (H_2_O_2_) that contributes to an increase in the steady-state concentrations of ROS within both the mitochondrial matrix and the cytosol. These released H_2_O_2_ and O_2_*^−^ are carried to the cytoplasm via voltage-dependent anion channels and, ultimately, lead to the oxidation of cytoplasmic proteins. The chronic exposure of ROS to cells can result in oxidative damage to mitochondrial and cellular proteins, lipids, and nucleic acids; and the acute exposure to ROS can inactivate the TCA-cycle aconitase and the iron-sulfur centers of ETC at complexes I, II and III, resulting in a shutdown of mitochondrial energy production [64]. Thus, mitochondria are critical in the metabolism of all mammalian cells, and abnormalities in mitochondrial structure and function may lead to age-related diseases, such as asthma.

## 5. Aging and Asthma

As earlier mentioned, asthma is known to be common in persons from childhood through middle age. However, several recent reports suggest that aged people are also affected [66-70] with asthma, although it is generally under diagnosed. Viral respiratory illnesses, including influenza, the common cold virus, and tobacco smoke are the main triggers for asthma in aged persons. Further, in children and middle-age persons, factors that commonly induce asthma, such as dust mites, pet dander, pollen, and molds, also affect aged people.

**Figure 3:**
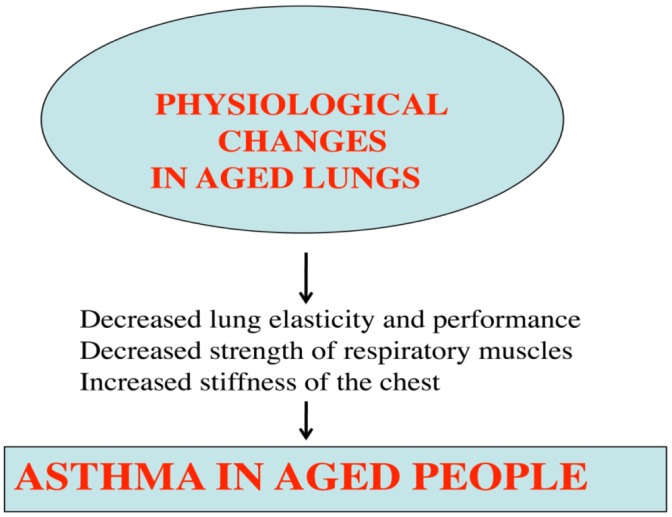
Age-dependent physiological changes in asthmatic persons. (1) Lung elasticity and performance are decreased; (2) the strength of respiratory muscle is decreased; (3) and the stiffness of chest is increased.

Lack of exercise or of healthy foods may also contribute to asthma in aged people. Further, several morphological and physiological abnormalities have been reported in the lungs of aged persons, including decreased mucociliary function, dilatation of air spaces, loss of elastic recoil, diminished diffusion capacity, and the presence of low grade inflammation of the respiratory tract [71] (Figure 3).

**Figure 4:**
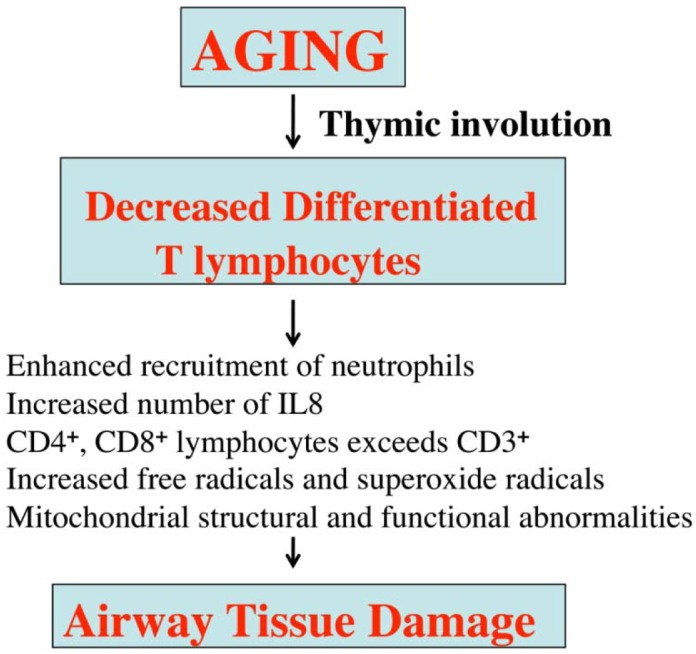
Age-related changes in T lymphocytes and asthma. Differentiated T lymphocytes decrease in aged people and in several T lymphocyte functions are abnormal, including: (1) enhanced recruitment of neutrophils; (2) increased number of IL8; (3) CD4+, CD8+ lymphocytes exceeds the CD3+; and (4) increased free radical production of T lymphocytes are impaired, leading to the susceptibility of asthma in aged persons.

The declining immune system in aged people has been identified as the major cause of several age-related immune diseases, including asthma. T-lymphocytes have reported to be impaired in aged people, in addition to important T-lymphocyte functions, such as cell-mediated immune responses [51]; CD4+ lymphocytes and CD8+ lymphocytes are higher in aged people than in young individuals, which may lead to reduced responsiveness of T lymphocytes and the consequent aggravation of inflammatory factors (Figure 4). In addition, the functions of several inflammatory cells, including mast cells, basophils, macrophages, neurophils, eosinophils, and epithelial cells, have been reported to be abnormal in aged people [66]. Further, neutrophils and IL8 are significantly elevated in this age group [66] (Figure 4).

Overall, structural and functional abnormalities of T lymphocytes may be responsible for the vulnerability of aged people to asthma.

## 6. Mitochondrial Dysfunction, Oxidative Stress, and Asthma

Multiple lines of evidence suggest that environmental pollutants and oxidants induce oxidative damage in mitochondria of in airway epithelial cells [73,74]—an inducement that ultimately triggers asthma. First, the exposure of oxidants and allergens induces airway inflammation, resulting in the release of pro-inflammatory mediators, including histamine and leukotrienes. These mediators recruit or activate various types of inflammatory cells, including eosinophils, neutrophils, lymphocytes, macrophages, and platelets [75]. In turn, these inflammatory cells release various reactive free radicals, such as superoxide anions, hydroxyl radicals, hydrogen peroxide, and nitric oxide, and they damage surrounding epithelial cells in the airway. Second, the asthmatic epithelium is known to be abnormally susceptible to apoptosis induced by oxidants, possibly due a lack of endogenous protective factors [73,74]. Third, increased numbers of mitochondria have been observed in the bronchial epithelium of asthmatic mouse models and of asthmatic children [76,77]. Fourth, ATP levels and mitochondrial abnormalities, including decreased cytochrome *c* oxidase activity and mRNA expression, were found to be decreased in the lungs of asthmatic mice [10]. Fifth, increased ultrastructural changes in mitochondria, such as the loss of cristae and mitochondrial swelling, have been found in an asthmatic mouse model [10]. Sixth, Aguilera-Aguirre *et al*. [11] recently found pre-existing mitochondrial dysfunction involved in the inflammation of the airways of allergic patients. In that study, nine oxidatively damaged, mitochondrial respiratory chain complex proteins were found after cellular oxidative insult induced by the exposure to a ragweed pollen extract. Seventh, several oxidative stress studies revealed that endogenous antioxidant enzymes were decreased in the peripheral tissues of asthmatic adult patients relative to the control subjects, indicating that mitochondrial dysfunction and oxidative stress is present in asthmatic adults [78-82]. Overall, these findings suggest that pre-existing mitochondrial dysfunction that is induced by oxidant environmental pollutants may be responsible for airway inflammation in asthmatic patients.

### 6.1. Oxidative Stress in Humans with Asthma

Recent studies of mitochondria dysfunction and oxidative stress using peripheral cells and tissues from asthmatic patients and control subjects suggest that antioxidant enzymes are in lower levels in asthmatic patients [78-85].

Comhair *et al*. [79] measured serum superoxide dismutase (SOD) activity and proteins, the glutathione peroxidase/glutathione antioxidant system, and oxidatively modified amino acids in adult subjects with asthma and healthy control subjects. They found SOD activity, but not manganese superoxide dismutase (Mn-SOD) or copper- and zinc-containing superoxide dismutase (Cu, Zn-SOD) protein, was lower in asthmatic serum as compared with control subjects, and activity loss was significantly related to airflow constriction. Further, serum SOD activity inversely correlated with circulating levels of 3-bromotyrosine, a post-translational modification of proteins produced by the eosinophil peroxidase system of eosinophils. These findings are consistent with greater oxidant stress found in asthmatic patients leading to greater inactivation of SOD, which may increase inflammation and progressive airflow obstruction.

To determine the antioxidant response of the respiratory epithelium to airway inflammation that characterizes asthma, De Raeve *et al*. [80] quantified-Cu, Zn-SOD, MnSOD, catalase, and glutathione peroxidase in bronchial epithelial cells of asthmatic and healthy control subjects. Although catalase and glutathione peroxidase in bronchial epithelium of asthmatics were similar to control SOD activity in asthmatics not on inhaled corticosteroid was lower than asthmatics on inhaled corticosteroid and controls. However, Mn-SOD and Cu, Zn-SOD mRNA, and protein levels did not change in the asthmatic subjects, nor in the control subjects. Importantly, Cu, Zn-SOD-specific activity in asthmatics not on inhaled corticosteroid was less than that in the asthmatic on inhaled corticosteroid and control subjects.

Wu *et al*. [81] determined the connection between eosinophil activation and tissue injury in asthmatic subjects. Using mass spectrometry, they determined that 3-bromotyrosine serves as a specific “molecular fingerprint” for proteins modified through the eosinophil peroxidase-hydrogen peroxide system, in the presence of plasma levels of halides. They applied a localized allergen to model the effects of eosinophils and brominating oxidants in human lung injury. Endobronchial biopsy specimens from the allergen-challenged asthmatic, but not healthy control, subjects exhibited significant enrichments in eosinophils and eosinophil peroxidase. Baseline levels of 3-bromotyrosine in bronchoalveolar lavage proteins from those asthmatic subjects who were mildly allergic were but not statistically significantly elevated over those levels in control subjects. After exposure to the allergen challenge, lung segments from the asthmatic subjects, but not healthy control subjects, exhibited a >10-fold increase in BAL 3-bromotyrosine content, but only a two-to threefold increase in 3-chlorotyrosine, a specific oxidation product formed by neutrophil- and monocyte-derived myeloperoxidase. These results indicated reactive brominating species as a distinct class of oxidants formed *in vivo*. This study concluded eosinophil peroxidase is a potential therapeutic target for allergen-triggered inflammatory tissue injury in humans.

Thomassen *et al*. [82] studied the *in vivo* effects of a localized allergen challenge on airway nitric oxide levels and the activation of a transcription factor. They found increased nitric oxide (NO) in the airway in the asthmatic subjects but not in the control subjects. The increased NO in the asthma subjects was associated with an increase in inflammatory cytokines, GM-CSF, and macrophage inflammatory protein-1 in the epithelial lining fluid and eosinophilic infiltrate in bronchoalveolar lavage fluid (BALF) and biopsy specimens. To investigate the mechanisms of cytokine gene expression, Thomassen *et al*. evaluated the activation of the transcription-factor activator protein-1 and the nuclear factor-kappaB (NF-kappaB) in cells from the BALF. The activator protein-1 was not activated before or after the local allergen challenge. In contrast, NF-kappaB activation was less in the BALF cells from asthmatic subjects with increased NO in comparison to control subjects.

Fitzpatrick *et al*. [83] measured antioxidant enzymes in the bronchoalveolar lavage from 65 children with severe asthma, including 35 children with baseline airway obstruction. Control data were obtained from six children with psychogenic cough or vocal cord dysfunction who were undergoing diagnostic bronchoscopy and from 35 healthy adult controls. GSH, GSSG, and other determinants of airway oxidative stress–including glutathione S-transferase, glutathione reductase, glutathione peroxidase, malondialdehyde, 8-isoprostane, and H_2_O_2_–were measured in epithelial lining fluid. The redox potential in the epithelial lining fluid was calculated from GSH and GSSG with the Nernst equation. Compared with both groups of controls, subjects with severe asthma had lower airway GSH and increased GSSG, even though there were no differences in GST, and GPx activities between both control groups and the asthmatic group. This was accompanied by increased malondialdehyde, 8-isoprostane, and H_2_O_2_ concentrations in the asthmatic children's epithelial lining fluid. This group also exhibited increased biomarkers of oxidant stress in the epithelial lining fluid that are associated with increased formation of GSSG and a shift in the GSH redox potential toward a more oxidized state.

Comhair *et al*. [84] showed that extracellular gluthione peroxidase (eGPx) is higher in the airways of adult asthmatic subjects than in those of healthy controls and that the source for the increased eGPx was bronchial epithelial cells. The eGPx mRNA in bronchial epithelial cells increased eightfold after the exposure to ROS and glutathione, the latter which is an essential cofactor in eGPx activity. Alterations in intracellular and extracellular oxidized and reduced glutathione were temporally associated with the increase of eGPx, further supporting the involvement of redox mechanisms in gene expression. The overexpression of superoxide dismutase, but not catalase, inhibited the induction of eGPx and identified superoxide as a key intermediary. The eGPx mRNA half-life was not affected by ROS, suggesting a transcriptional mechanism for eGPx regulation. Fusion genes of deletion fragments of the eGPx gene 5′ flanking region driving a reporter gene conclusively identified the ROS-responsive region, which contained the consensus DNA binding site for the redox-regulated transcription factor, activator protein 1.

Using gas chromatography-mass spectrometry, MacPherson *et al*. [85] found a 10-fold increase in 3-nitrotyrosine content, a global marker of protein modification, in proteins recovered from the bronchoalveolar lavage of severe asthmatic patients compared with nonasthmatic subjects. Parallel gas chromatography-mass spectrometry analyses of bronchoalveolar lavage proteins for 3-bromotyrosine and 3-chlorotyrosine (selective markers of eosinophil peroxidase and myeloperoxidase-catalyzed oxidation, respectively) showed a dramatic preferential formation of 3-bromotyrosine in the asthmatic subjects compared to the nonasthmatic subjects. Bronchial tissue from individuals who died of asthma exhibited the most intense anti-3-nitrotyrosine immunostaining in epitopes that colocalized with eosinophils. These studies identified eosinophils as a major source of oxidants in asthmatic subjects.

Taken together, these studies suggest that antioxidant approaches in asthmatic persons may be useful to treat them.

### 6.2. Oxidative Stress in Induced Mouse Models of Asthma

Researchers do not know precise genetic factors underlying asthma, and without such information, it is not possible to create genetically engineered animal models to study asthma. However, several groups have developed induced asthmatic mouse models in order to study mechanisms of allergic asthma [10,76,77,86,87]. In most of these mouse models, mice are sensitized to an allergen with alum as an adjuvant, and then, over a period of 1–9 days for acute models and 5–9 weeks for chronic models, the mouse are challenged with an allergen via their airways [87]. These mice developed such changes as airway inflammation, reversible airway obstruction, eosinophilia in airway tissue, increased CD4+ lymphocytess, increased CD8+ lymphocytes, mucus hyperproduction, increased allergen-specific IgE, increased Th2 cytokines, early- and late-phase responses, and mitochondrial structural and functional abnormalities [10,76,77,88,89]. Although these models do not develop all of the asthmatic features known to plague humans with asthma, they do develop several of these asthmatic features, making them good models for the study of asthma progression.

Mabalirajan *et al*. [10] developed a mouse model that is allergic to OVA. This model exhibits several features of mitochondrial dysfunction, such as reduction of cytochrome *c* oxidase activity in lung mitochondria, reduction in the expression of subunit III of cytochrome *c* oxidase in the bronchial epithelium, the appearance of cytochrome *c* in the lung cytosol, decreased levels of ATP in the lungs, reduced expression of 17 kDa of complex I in the bronchial epithelium, and ultrastructural changes in mitochondria, such as swelling of mitochondria and the loss of cristae. These features suggest that changes in mitochondrial structure and mitochondrial dysfunction are associated with allergic asthma.

Park *et al*. [90] studied the relationship between increased pulmonary oxidative stress and features of asthma during an allergic inflammatory response. Asthmatic parameters were measured at 9 time points, starting from the first day of the challenge with and without antioxidant treatment. Bronchoalveolar lavage fluid cells, histopathologic features, and airway hyper-responsivenesswere also measured at the same time points. Park *et al*. found that the oxidized glutathione ratio was reduced from immediately after the OVA challenge to day 1. The ratio then rapidly recovered to normal at day 2. At day 3, the inflammatory cells in the BALF reached a maximum level and then progressively decreased. However, histopathologic examination revealed that substantial airway inflammation persisted through day 28. These results indicate that increased oxidative stress in the lung precedes other key phenotypes of allergic airway disease.

Konga *et al*. [91] studied the effects from computer-printer emissions and cigarette smoke on the structure and function of mitochondria in asthmatic mice by measuring the levels of ROS, lipid peroxides, and glutathione, and by measuring the activities of isocitrate dehydrogenase, alpha-ketoglutarate dehydrogenase, succinate dehydrogenase, malate dehydrogenase, complexes I to IV, and cytochrome oxidase activity. In addition, oxidative phosphorylation was evaluated to assess the functional capacity of mitochondria. These researchers found highly elevated levels of ROS and lipid peroxides, and reduced levels of mitochondrial enzymes in the asthmatic mice exposed to only the tobacco smoke and in those exposed to both printer emissions and the tobacco smoke. However, the asthmatic mice exposed to only printer emissions exhibited only slight increases in the parameters studied. Based on these results, Konga *et al*. [91] concluded that printer emissions exert a synergistic effect in the presence of tobacco smoke and that printer emissions increase damage to the lung mitochondria by disrupting the structural and functional integrity of the mitochondrial membrane.

Bharadwaj and Agrawal [92] studied dendritic cells to identify morphological changes in OVA-sensitized mice after they were exposed to allergens. They found that the dendritic cells in the lungs of challenged mice were more mature owing to their pronounced dendrites than were the dendritic cells in the lungs and spleen of control mice that were treated with PBS. The presence of large numbers of well-developed mitochondria and rough endoplasmic reticulum in myeloid dendritic cells from both lungs and spleen of OVA-sensitized and challenged mice indicate greater functional activity. Additionally, dendritic cells from the OVA-sensitized and challenged mice also exhibited more fat and glycogen stores than the control mice. These findings suggest that morphological features may be indicate mature, fully functioning dendritic cells and and mitochondrial abnormalities in the lungs of allergic asthmatic mice.

Using 2-dimensional polyacrylamide gel electrophoresis and liquid chromatography-tandem mass spectrometry techniques, Zhang *et al*. [93] studied the impact of the thiol antioxidant, N-acetylcysteine, on protein expression in an OVA mouse model. They found that several oxidantive stress proteins (e.g., chitinase 3-like protein, acidic mammalian chitinase pulmonary surfactant-associated protein D, resistin-like molecule alpha and haptoglobin alpha-subunit) were significantly greater in the BALF of the OVA-challenged mice, compared to the control group. These findings suggest that oxidative stress proteins may play an important role in the pathogenesis of asthma [93].

Inoue and colleagues [94] studied the role of peroxiredoxin 1 (Prx 1), a ubiquitous antioxidant enzyme in mouse allergic inflammation. They sought to determine whether endogenous Prx I protects against allergic asthma traits in Prx-1 knockout (−/−) mice. Prx 1 (−/−) and wild-type mice were immunized with OVA plus aluminum potassium sulfate, and subsequently challenged with OVA. Twenty-four hours after the last OVA challenge, the influx of leukocyte influxs, including eosinophils, into BALF was significantly greater in the Prx 1 (−/−) mice compared to the influx in the wild-type mice. In contrast, when the wild-type mice were immunized with OVA and complete Freund's adjuvant, the opposite phenomenon was observed: in the Prx 1(−/−) mice, in the presence of OVA/alum, the infiltration of peribronchial inflammatory leukocytes, the resistance of the cholinergic airway, and the expression of interleukin (IL)-2 were significantly greater and the interferon-gamma was significantly less than in the wild-type mice. These findings suggest that endogenous Prx 1 protects against allergen-related Th2-type airway inflammation and hyper-responsiveness via the suppression of IL-2 in lungs and the regulation of the Th1/Th2 balance [94].

Overall, these findings suggest that mitochondrial structural and functional abnormalities are associated with allergic asthma. However, without an animal model that faithfully mimics the allergic features of asthmatic humans, it is difficult elucidating mitochondrial abnormalities in asthmatic patients.

## 7. Therapeutic Approaches to Asthma

Several therapeutic approaches have been developed to reduce and/or prevent the symptoms of allergies and asthma in humans: the control and reduction of allergens in the environment, oral antihistamines, decongestants, intranasal corticosteroids, mast cell stabilizers, and immuno- and antioxidant- therapies

### 7.1. Antioxidant Approaches: Evidence from Experimental Rodent Models of Asthma

Increasing evidence suggests that mitochondrial dysfunction and oxidative stress are associated with asthma, but the precise nature of this association is unclear. Several groups found decreased mitochondrial dysfunction and oxidative stress in asthmatic mice treated with antioxidants [95-105]. For example, using a C57BL/6 mouse model of allergic asthma, Lee *et al*. [95] studied the role of antioxidants in regulating the receptor activator of NF-kappaB expression. They evaluated the effects of the vascular growth factor receptor inhibitor and L-2-oxothiazolidine-4-carboxylic acid (a prodrug of cysteine), on the receptor activator of NF-kappaB mRNA expression. The mice developed pathophysiological features of asthma in the lungs: increased expression of the receptor activator of NF-kappaB mRNA, an increased number of inflammatory cells in the airways, increased vascular permeability, and increased levels of vascular endothelial growth factor receptor. In allergen-induced asthmatic lungs of the C57BL/6 mice, L-2-oxothiazolidine-4-carboxylic acid and a vascular endothelial growth factor receptor inhibitor markedly reduced the levels of plasma extravasation and vascular endothelial growth factor receptors. Seventy-two hours after the mice inhaled, expression of the receptor activator of NF-kappaB mRNA was reduced. These results indicate that the L-2-oxothiazolidine-4-carboxylic acid and the vascular endothelial growth factor receptor inhibitor may be associated with the regulation of vascular permeability. Further, these results suggest that the vascular endothelial growth factor receptor may regulate the receptor activator of NF-kappaB expression. These findings provide a crucial molecular mechanism that can justify the use of antioxidants to prevent and/or to treat asthma and other airway inflammatory disorders.

Using a C57BL/6 mouse model of allergic asthma, Lee *et al*. [96] studied the effects of antioxidants on the regulation of IL-18 expression. They found increased levels of ROS production in cells from broncho-alveolar lavage fluids and that the administration of L-2-oxothiazolidine-4-carboxylic acid or alpha-lipoic acid reduced the increased levels of ROS, the expression of IL-18 mRNA and protein, airway inflammation, and bronchial hyper-responsiveness. They also found that antioxidants down-regulated nuclear factor-kappaB and that antioxidants may reduce IL-18 expression in asthma mice by inhibiting NF-kappaB activity.

Castro *et al*. [97] investigated the effect of the antioxidant butylated hydroxyanisole (BHA) on respiratory syncytial virus-induced lung inflammation, and airway hyper-reactivity in BALB/c mice. The mice were infected with plaque-forming units of a respiratory syncytial virus, in the presence and in the absence of orally administered BHA. BHA significantly attenuated respiratory syncytial, virus-induced lung oxidative stress, indicated by a decrease in malondialdehyde and 4-hydroxynonenal content, in the broncho-alveoar lavage of the respiratory syncytial, virus-infected mice. BHA also reduced respiratory syncytial virus-induced clinical illness and body weight loss, and attenuated respiratory syncytial virus-induced airway hyper-reactivity. This study concluded that modulation of oxidative stress may be a novel pharmacologic approach to ameliorate respiratory syncytial virus-induced acute lung inflammation.

Mabalirajan *et al*. [98] studied the effects of Vit-E in an experimental allergic BALB/c mouse model. The OVA-sensitized and challenged mice exhibited characteristic features of asthma, such as airway hyper-responsiveness, airway inflammation, and airway remodeling. In addition, they showed increased expression in the metabolites of 12/15-lipoxygenase, a reduction in the activity and expression of the third subunit of mitochondrial cytochrome-c oxidase, and increased cytochrome c in lung cytosol, all of which indicate that OVA sensitization may cause mitochondrial dysfunction in mice. Vit-E was administered orally to these mice and levels of 12/15-LOX expression, key mitochondrial functions, and ultrastructural changes of mitochondria in bronchial epithelia were measured. Vit-E treatment resulted in the reduction of key mitochondrial dysfunctions and the alleviation of asthmatic features, including reduced airway hyper-responsiveness; Th2 responses, including IL-4, IL-5, IL-13, and OVA-specific IgE; reduced eotaxin; airway inflammation; expression and metabolites of 12/15-LOX in lung cytosol; lipid peroxidation; nitric oxide metabolites in the lung; restored activity and expression of the third subunit of cytochrome-c oxidase in lung mitochondria and bronchial epithelia, respectively; reduced appearance of cytochrome c in lung cytosol; and restored mitochondrial ultra-structural changes in the bronchial epithelia.

Mabalirajan *et al*. [99] studied the effects of esculetin, a plant-derived coumarin and immunomodulator, to restore anti-asthma properties and mitochondrial dysfunction and structural changes in a mouse model of experimental asthma. Treatment with esculetin reduced airway hyper-responsiveness, Th2 response, lung eotaxin, BALF eosinophilia, airway inflammation, and OVA-specific IgE. Esculetin reduced the metabolites of 15-lipoxygenase and lipid peroxidation, the latter which is an essential prerequisite for mitochondrial dysfunction. Interestingly, esculetin restored the activity of cytochrome c oxidase of the ETC in lung mitochondria and also restored the expression of the third subunit of cytochrome c oxidase in the ETC of the bronchial epithelium. It reduced the level of cytochrome c and caspase 9 activity in the lung cytosol, and restored mitochondrial structural changes and ATP levels in the lung. In addition, esculetin reduced subepithelial fibrosis and TGF-beta 1 levels in the lung. These results suggest that esculetin not only restores mitochondrial dysfunction and structural changes but also alleviates asthmatic features.

Using an OVA-induced allergic mouse model of asthma, Lee *et al*. [100] investigated the suppressive effects of resveratrol on asthmatic parameters, including cytokine release, eosinophilia, airway hyper-responsiveness, and mucus hypersecretion. Resveratrol significantly inhibited increases in Th2 cytokines, such as IL-4 and IL-5, in plasma and BALF and also suppressed airway hyper-responsiveness, eosinophilia, and mucus hypersecretion. The efficacy of resveratrol was similar to that of dexamethasone, a glucocorticoid that was used as a positive control. These results suggest that resveratrol may have applications in the treatment of bronchial asthma.

Using gene expression analysis, Dittrich *et al* [101] studied the genes and proteins involved in allergic airway disease, in asthma mice. They found increased expression of two antioxidant enzymes, glutathione peroxidase-2 and glutathione-S-transferase omega 1-1, in two mouse strains after allergic airway disease was induced and localized in lung epithelial cells. Mice with targeted disruption of the glutathione peroxidase-2 gene showed significantly enhanced airway inflammation compared to the sensitized and challenged wild-type mice. These data indicate that genes encoding the antioxidants glutathione peroxidase-2 and glutathione-S-transferase omega 1-1 are genes expressed upon the induction of allergic airway inflammation, independently of allergic susceptibility.

Chang *et al*. [102] studied the effects of vitamin C on OVA-sensitized and challenged mice. They found that dexamethasone treatments and a diet supplemented with high doses of vitamin C significantly decreased the infiltration of eosinophilia into BALF. A diet supplemented with a high dose of vitamin C significantly increased the ratio of interferon-gamma/interleukin-5 cytokines, suggesting that high doses of vitamin C may attenuate allergic inflammation *in vivo* by modulating the Th1/Th2 balance toward the Th1 pole during the Th2-skewed allergic airway inflammation and decreasing eosinophilic infiltration into BALF.

Ahmad *et al*. [103] studied endogenous asymmetric dimethylarginine and peroxynitrite in murine mouse models of an allergic airway inflammation that resembles asthma. Asymmetric dimethylarginine levels and nitrosative stress positively correlated with cytosol and mitochondria during allergic airway inflammation. This was associated in bronchial epithelia with an increased expression of protein-arginine methyltransferase-2 and a reduced expression of dimethylarginine dimethylaminohydrolase-2. Increased nitrotyrosine was similarly localized to the bronchial epithelium, as well as in the infiltrated inflammatory cells. Administration of L-arginine–which was expected to compete with asymmetric dimethylarginine and to reverse the uncoupling/inhibition of nitric oxide synthase restored normal asymmetric dimethylarginine metabolism and restored a reduction innitrosative stress in the lung. Because dimethylarginine dimethylaminohydrolase-2 function is known to be negatively related to oxidative stress, this may represent a feed-forward loop effect.

Okamoto *et al*. [104] investigated the role of antioxidants in airway hyper-responsiveness to acetylcholine using young asthmatic mice. The mice were fed either a normal diet, an alpha-tocopherol-supplemented diet, or a probucol-supplemented diet 14 days before the first sensitization. They were immunized with an antigen at 12-day intervals. Starting from 10 days after the second immunization, they were exposed to the antigen three times every fourth day using an ultrasonic nebulizer. Twenty-four hours after the last antigen inhalation, airway responsiveness to acetylcholine was measured and BALF was collected. They found significantly decreased levels of IL-4 and IL-5 in the BALF of the alpha-tocopherol-supplemented mice. The serum IgE level was decreased in probucol-supplemented mice. Airway hyper-responsiveness to acetylcholine was repressed in the antioxidant-supplemented mice. These findings suggest that alpha-tocopherol and probucol suppress allergic responses in asthmatic mice.

In a recent study of antioxidants, Mehta *et al*. [105] investigated the efficacies of choline and the antioxidant alpha-lipoic acid in a rodent model of allergic asthma. In the mice receiving choline and alpha-lipiic acid, these researchers found significantly decreased total lymphocyte counts, total eosinophil counts, eosinophil peroxidase activity maloialdehyde in the BALF [105].

Overall, these studies suggest that antioxidant treatments are beneficial to asthmatic experimental rodents and may have potential use as treatment for asthmatic patients.

### 7.2. Antioxidant Approaches: Evidence from Asthmatic Humans Treated with Antioxidants

As described above, mitochondrial dysfunction and oxidative stress are key factors that are involved in humans with allergic asthma and also in mouse models of allergic asthma. It is reasonable to treat asthmatic humans with antioxidants to decrease the effects of oxidants. There are several antioxidants and amino acid substrate, L-arginine available, including endogenous metobolites (glutathione, *N*-actylcysteine, heme oxygenase 1), natural antioxidants and other nutrients (vitamin C, E, co-enzyme Q10, curcumin, alpha lipoic acid, fish oil), and herbal molecules and polyphenols (esculitin, sulforaphane, PG102, resveratrol, epigallocatechin 3 gallate, caffeic acid phenethyl ester). Currently, these antioxidants have been tested in experimental mouse models of asthma and cell lines from asthmatic humans, and in clinical trials of asthmatic humans [106].

Recently, Gazdik *et al*. [107,108] measured coenzyme Q10 levels in the plasma and whole blood of asthmatic humans and found decreased levels of coenzyme Q10. They also measured coQ10, alpha-tocopherol, and beta-carotene in the plasma and whole blood in patients with bronchial asthma and in healthy control subjects [107]. They found significantly decreased levels of coQ10 and alpha-tocopherol in the plasma and whole blood of bronchial asthmatic humans compared to healthy subjects, indicating oxidative damage in asthmatic humans. The same research group [109] studied the effects of coQ10 on asthma patients by supplementing coQ10 in their diets. In a randomized clinical study of 41 bronchial asthma patients (13 males, 28 females), ages 25–50 years, all suffered from persistently mild-to-moderate asthma. They were divided into two subject groups: one group received a standard anti-asthmatic therapy and was clinically stabilized, and the second group received the same standard anti-asthmatic therapy plus the antioxidant coQ10 as Q-Gel (120 mg) + 400 mg alpha-tocopherol and 250 mg of vitamin C per day. The groups were crossed over at 16 weeks, for a total duration of 32 weeks. The asthmatic patients fed a diet supplemented with CoQ10 had reduced levels of corticosteroids compared to the levels of corticosteroids in healthy subjects, supporting the hypothesis that antioxidants are useful in reducing oxidative stress in asthmatic patients [109].

Several studies recently assessed the *N*-acetylcysteine in reducing oxidative stress in asthmatic humans [110-113]. The outcomes of these studies have been inconclusive, particularly in terms of understading the upper airway epithelium. Recently, Moradi *et al*. studied the effects of NAC (IV NAC in 150 mg/kg on day 1, followed by 50 mg/kg/day for three more days) on 27 patients with acute lung injury considering the glutathione-*S*-transferase genetic variations, as an important enzyme contributing in oxidative stress pathways. NAC was found to improve oxygenation and to decrease the mortality rate in the treated asthmatic subjects compared to the control subjects.

Several other studies assessed the effects of natural antioxidants (e.g., vitamin C, E, beta carotene) on asthmatic humans, and found that diets supplemented with them did not decrease or otherwise affect asthmatic features [114-118]. However, Papas *et al*. [119] evaluated the safety of a novel micellar formulation (CF-1) of fat-soluble nutrients and antioxidants. Ten subjects, ages 8 to 45 years old, were given orally 10 ml of the CF-1 formulation daily for 56 days after a 21-day washout period in which subjects stopped supplemental vitamin use, except for a standard multivitamin. Plasma obtained at -3, 0, 1, 2, 4, and 8 weeks was assayed for beta-carotene, gamma-tocopherol, retinol, and CoQ10, as well as for safety parameters. In addition, pulmonary function was measured and induced sputum was assayed for markers of inflammation and quantitative bacterial counts prior to and during dosing. Supplementation with CF-1 significantly increased beta-carotene levels at all dosing time points compared to levels at screening and baseline. In addition, gamma-tocopherol and CoQ10 significantly increased from baseline in all subjects. The novel CF-1 formulation safely and effectively increased plasma levels of important fat-soluble nutrients and antioxidants. In addition, improvements in antioxidant plasma levels were associated with reductions in airway inflammation in CF patients.

Overall, the natural antioxidants and nutrients did not show strong beneficial effects in patients with asthma [114-118]. One possible reason for this limited success is that natural antioxidants may not reach sites of free radical production effectively, and they may scavenge free radicals and decrease oxidative damage in asthmatic patients. Targeted antioxidants to mitochondria may decrease mitochondrially derived oxidative stress much more effectively than natural antioxidants [120].

## 8. Mitochondria-Targeted Antioxidants: Potential Therapeutic Molecules for Asthma

Recently, antioxidants to prevent and to treat mitochondria in patients with mitochondrial diseases, including asthma, has received much attention, especially because antioxidant approaches seem to have few or no adverse effects. However, they may be prevented from doing so because naturally occurring antioxidants, such as vitamins E and C, may not reach the relevant sites of free radical generation, especially if mitochondria are the primary source of ROS [120]. To overcome this problem and to better assess whether antioxidant approaches may be a productive treatment for asthma, a better system to deliver antioxidants to lung mitochondria of asthmatic persons is needed.

**Figure 5:**
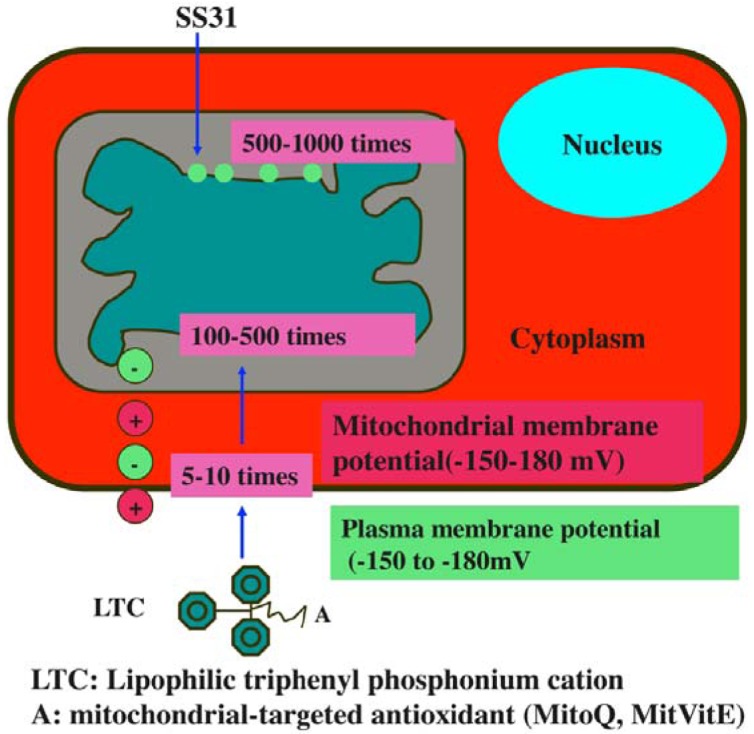
Mitochondria-targeted antioxidants as therapeutic agents for asthma. A generic mitochondrial-targeted antioxidant is shown constructed by the covalent attachment of an antioxidant molecule to the lipophilic triphenylphosphonium cation. Antioxidant molecules accumulate 5–10 fold in the cytoplasm, which is driven by plasma membrane potential, and then further accumulate several hundred-fold in the mitochondria. Mitochondria-targeted antioxidants neutralize free radicals and reduce mitochondrial toxicity. SS31 peptide (or Szeto-Schiller31 peptide) is cell-permeable, mitochondria-targeted antioxidant protects mitochondria from oxidative damage. The structural motif of these SS31 centers on alternating aromatic residues and basic amino acids and SS31 has a sequence motif that allows it to target mitochondria several hundred fold more than natural antioxidants. Once SS31 reach mitochondria, it rapidly neutralize free radicals and decrease mitochondrial toxicity. These targeted antioxidants to mitochondria are promising agents that may protect inflammatory cells in patients with asthma.

Considerable progress has been made in the last seven years, in developing mitochondria-targeted antioxidants, such as triphenylphosphonium-based antioxidants (MitoQ, MitoVitE, MitoPBN) and cell-permeable, small peptide-based antioxidants (SS02, SS31, SS19, and SS20) [64,121-123] (Figure 5). The efficacies of these antioxidants are being tested in cell and animal models of neurodegenerative diseases [124-125], but not yet in patients with allergies or asthma.

### 8.1. Cell-Permeable, Small Peptide Antioxidants

Recently, Szeto and Schiller developed a series of four small, cell-permeable, mitochondria-targeted antioxidant peptides (Szeto-Schiller or SS peptides) that protect mitochondria from oxidative damage [122,123]: (1) SS-01 H-Tyr-D-Arg-Phe-Lys-NH_2_; (2) SS-02 H-Dmt-D-Arg-Phe-Lys-NH_2_; (3) SS-31 H-D-Arg-Dmt-Lys-Phe-NH_2_; and (4) SS-20 H-Phe-D-Arg-Phe-Lys-NH_2_. Mechanistically, the SS31 peptide targets the inner mitochondrial membrane, due to the electrostatic attraction between these cationic peptides (positive charge) and the highly anionic cardiolipin molecules (negative charge) of the inner mitochondrial membrane. Further, SS31 has a dimethyltyrosine residue, allowing SS31 to scavenge oxyradicals and inhibit linoleic acid and low density lipoprotein oxidation. By reducing mitochondrial ROS, SS31 was able to prevent the opening of the MPT pore, prevent mitochondrial swelling, and reduce cytochrome c release in response to a high Ca^2+^ overload [122,123].

An overload of intracellular calcium can also lead to an increase in mitochondrial ROS and an opening of the mitochondrial permeability transition (MPT) pore [64]. However, by reducing mitochondrial ROS, scavenging SS31 inhibited the opening of the MPT, which prevented mitochondrial swelling and reduced the release of cytochrome *c* in response to calcium overload [120]. These results support the hypothesis that ROS potentiates the MPT pore via oxidation of the adenine nucleotide translocator. SS31 was found to prevent the MPT from opening, which led to a minimization of MPT-induced ROS accumulation and also led to a reduction in oxidative damage, in mitochondria [122,123]. Recently, the efficacy of SS31 in terms of its ability to protect neurons has been tested, using ALS transgenic mice. When they were treated with SS31, the mice exhibited extended lifespan compared to untreated mice, suggesting that SS31 is neuroprotective and may neutralize mitochondrially generated free radicals, decrease oxidative damage, and boost mitochondrial function [124]. Further, in studies of Parkinson's disease that used experimental MPT mice, researchers found that SS31 decreases mitochondrial swelling and toxicity, and prevents dopaminergic cell death [125].

More recently, using mouse neuroblastoma (N2a) cells and primary neurons from C57BL/6 mice, Reddy *et al*. [126] investigated the toxicity of herbicides, picloram and triclopyr, and protective effects of SS1 peptide against picloram and triclopyr toxicity. They measured total RNA content, cell viability and mRNA expression of endogenous levels of peroxiredoxins, neuroprotective genes (PGC1α, FOXO1, NMDA receptor) ETC genes in N2a cells treated with herbicides and SS31. Using primary neurons from C57BL/6 mice, Reddy *et al*. [126] studied neuronal survival in neurons treated with herbicides, in neurons pretreated with SS31 plus treated with herbicides, neurons treated with SS31 alone, and untreated neurons. They found significantly decreased total RNA content, and cell viability in N2a cells treated with herbicides compared to untreated N2a cells. Decreased mRNA expression of neuroprotective genes, and ETC genes in cells treated with herbicides was found compared to untreated cells. However, cells pretreated with SS31 prevented toxicity caused by herbicides. Immunofluorescence analysis of primary neurons revealed that decreased neuronal branching and degenerating neurons in neurons treated with picloram and triclopyr. However, neurons pretreated with SS31 prevented degenerative process caused by herbicides. Based on these results, they conclude that SS31 peptide appears to protect neurons from herbicide toxicity [126].

Recently, Manczak *et al*. [127] investigated the effects of the mitochondria-targeted antioxidants, MitoQ and SS31, and the anti-aging agent resveratrol on neurons from a mouse mode of Alzheimer's disease (AD) and N2a cells incubated with the amyloid-beta (Aβ) peptide. Using electron and confocal microscopy, gene expression analysis, and biochemical methods, they studied mitochondrial structure and function and neurite outgrowth in N2a cells treated with MitoQ, SS31, and resveratrol, and then incubated with Aβ. In N2a cells only incubated with the Aβ, they found increased expressions of mitochondrial fission genes, Drp1 and Fis1 and decreased expression of fusion genes, Mfn1, Mfn2 and Opa1 and also decreased expression of peroxiredoxins. Electron microscopy of the N2a cells incubated with Aβ revealed a significantly increased number of mitochondria. Biochemical analysis revealed that function is defective in mitochondria. Neurite outgrowth was significantly decreased in Aβ-incubated N2a cells, indicating that Aβ affects neurite outgrowth. However, in N2a cells treated with MitoQ, SS31, and resveratrol, and then incubated with Aβ, abnormal expression of peroxiredoxins and mitochondrial structural genes were prevented and mitochondrial function was normal; intact mitochondria were present and neurite outgrowth was significantly increased. In primary neurons from Aβ precursor protein transgenic mice that were treated with MitoQ and SS31, neurite outgrowth was significantly increased and cyclophilin D expression was significantly decreased. These findings suggest that mitochondria-targeted antioxidants, MitoQ and SS31 prevent Aβ toxicity [127].

Findings from these studies suggest that mitochondria-targeted antioxidants have great potential as a treatment for people with asthma. Further research is needed to evaluate the efficacies of mitochondrial-targeted antioxidants in experimental models of asthma, not only in experimental mouse models but also in nonhuman primate models (which is genetically closer to humans).

## 9. Conclusions and Future Directions

Recent genetic studies have revealed that multiple genetic loci are involved in the etiology of asthma, and recent advances in cellular, molecular, and animal model studies have revealed that several cellular events are involved in the progression of asthma, including (1) an imbalanced Th1/Th2 paradigm; (2) increased Th2 cytokines leading to the recruitment of inflammatory cells to the airway and the activation of those cells; (3) increased production of ROS and mitochondrial dysfunction in the activated inflammatory cells, leading to tissue injury in the bronchial epithelium; and (4) mitochondrial defects in allergic mice and asthmatic humans. Initial studies of mitochondrial dysfunction and oxidative stress revealed that mitochondrial oxidative stress is critically involved in asthma and may play a large role in the development of allergic asthma. Further, recent studies of antioxidants using experimental allergic asthmatic mouse models suggest that mitochondria-targeted antioxidants may be promising treatments for people with asthma. However, mitochondria-targeted antioxidants have not been tested as anti-asthmatic drugs. Investigation into these areas may improve our understanding of asthma and may help develop therapeutics of asthma.

## Abbreviations

AHR: airway hyper-responsiveness
ATP: adenosine triphosphate
DCs: dendritic cells
ETC: electron transport chain
LOX: lypoxygenase
MPT: mitochondrial permeability transition
mtDNA: mitochondrial DNA
OVA: ovalbumin
Prx1: peroxiredoxins 1
ROS: reactive oxygen species
TAC: tricarboxylic acid
Th1: T helper type 1
Vit-E: vitamin E
eGpx: extracellular glutathione peroxidase
GSH: glutathione
GST: glutathione-S-transferase
GSSG: glutathione disulfide
GPx: glutathione peroxidase
SS31: (Szeto-Schiller31)
mtDNA: mitochondrial DNA
BALF: bronchoalveolar lavage fluid

## References

[1] Busse W.W., Lemanske R.F. (2001). Asthma. N. Engl. J. Med..

[2] Lebowitz M.D., Barbee R., Burrows B. (1984). Family concordance of IgE, atopy, and disease. J. Allergy Clin. Immunol..

[3] Sarafino E.P., Goldfedder J. (1995). Genetic factors in the presence, severity, and triggers of asthma. Arch. Dis. Child..

[4] Zamel N. (1995). In search of genes of asthma on the island of Tristan da Cunha. Can. Resp. J..

[5] Heinzmann A., Deichmann K.A. (2001). Genes for atopy and asthma. Curr. Opin. Allergy Clin. Immunol..

[6] Van Eerdewegh P., Little R.D., Dupuis J., Del Mastro R.G., Falls K., Simon J., Torrey D., Pandit S., McKenny J., Braunschweiger K. (2002). Association of the ADAM33 gene with asthma and bronchial hyperresponsiveness. Nature.

[7] Heinzmann A., Thoma C., Dietrich H., Deichmann K.A. (2003). Identification of common polymorphisms in the mitochondrial genome. Allergy.

[8] Jones M., Mitchell P., Wang J.J., Sue C. (2004). MELAS A3243G mitochondrial DNA mutation and age related maculopathy. Am. J. Ophthalmol..

[9] Raby B.A., Klanderman B., Murphy A., Mazza S., Camargo C.A., Silverman E.K., Weiss S.T. (2007). A common mitochondrial haplogroup is associated with elevated total serum IgE levels. J. Allergy Clin. Immunol..

[10] Mabalirajan U., Dinda A.K., Kumar S., Roshan R., Gupta P., Sharma S.K., Ghosh B. (2008). Mitochondrial structural changes and dysfunction are associated with experimental allergic asthma. J. Immunol..

[11] Aguilera-Aguirre L., Bacsi A., Saavedra-Molina A., Kurosky A., Sur S., Boldogh I. (2009). Mitochondrial dysfunction increases allergic airway inflammation. J. Immunol..

[12] Holgate S.T., Davies D.E., Puddicombe S., Richter A., Lackie P., Lordan J., Howarth P. (2003). Mechanisms of airway epithelial damage: epithelial-mesenchymal interactions in the pathogenesis of asthma. Eur. Respir. J..

[13] Steinke J.W., Borish L. (2001). Th2 cytokines and asthma. Interleukin-4: its role in the pathogenesis of asthma, and targeting it for asthma treatment with interleukin-4 receptor antagonists. Respir. Res..

[14] Fanta C.H. (2009). Asthma. N. Engl. J. Med..

[15] Martinez F.D. (2007). Genes, environments, development and asthma: A reappraisal. Eur. Respir. J..

[16] Eisner M.D., Yelin E.H., Katz P.P., Earnest G., Blanc P.D. (2002). Exposure to indoorcombustion and adult asthma outcomes: environmental tobacco smoke, gas stoves, and woodsmoke. Thorax.

[17] Sarir H., Mortaz E., Karimi K., Kraneveld A.D., Rahman I., Caldenhoven E., Nijkamp F.P., Folkerts G. (2009). Cigarette smoke regulates the expression of TLR4 and IL-8 production by human macrophages. J. Inflamm (Lond).

[18] Thomson N.C., Spears M. (2005). The influence of smoking on the treatment response in patients with asthma. Curr. Opin. Allergy Clin. Immunol..

[19] Ober C., Hoffjan S., Hoffjan S. (2006). Asthma genetics 2006: The long and winding road to gene discovery. Genes Immun..

[20] Bouzigon E., Corda E., Aschard H., Dizier M.H., Boland A., Bousquet J., Chateigner N., Gormand F., Just J., Le Moual N., Scheinmann P., Siroux V., Vervloet D., Zelenika D., Pin I., Kauffmann F., Lathrop M., Demenais F. (2008). Effect of 17q21 variants and smoking exposure in early-onset asthma. N. Engl. J. Med..

[21] Raby B.A., Van Steen K., Lasky-Su J., Tantisira K., Kaplan F., Weiss S.T. (2009). Importin-13 genetic variation is associated with improved airway responsiveness in childhood asthma. Respir. Res..

[22] Sharma S., Murphy A.J., Soto-Quiros M.E., Avila L., Klanderman B.J., Sylvia J.S., Celedón J.C., Raby B.A., Weiss S.T. (2009). Association of VEGF polymorphisms with childhood asthma, lung function and airway responsiveness. Eur. Respir. J..

[23] Hunninghake G.M., Cho M.H., Tesfaigzi Y., Soto-Quiros M.E., Avila L., Lasky-Su J., Stidley C., Melén E., Söderhäll C., Hallberg J. (2009). MMP12, lung function, and COPD in high-risk populations. N. Engl. J. Med..

[24] Sharma S., Tantisira K., Carey V., Murphy A.J., Lasky-Su J., Celedón J.C., Lazarus R., Klanderman B., Rogers A., Soto-Quirós M. (2010). A role for Wnt signaling genes in the pathogenesis of impaired lung function in asthma. Am. J. Respir. Crit. Care Med..

[25] Verlaan D.J., Berlivet S., Hunninghake G.M., Madore A.M., Larivière M., Moussette S., Grundberg E., Kwan T., Ouimet M., Ge B. (2009). Allele-specific chromatin remodeling in the ZPBP2/GSDMB/ORMDL3 locus associated with the risk of asthma and autoimmune disease. Am. J. Hum. Genet..

[26] Halapi E., Gudbjartsson D.F., Jonsdottir G.M., Bjornsdottir U.S., Bjornsdottir U.S., Thorleifsson G., Helgadottir H., Williams C., Koppelman G.H., Heinzmann A. (2010). A sequence variant on 17q21 is associated with age at onset and severity of asthma. Eur. J. Hum. Genet..

[27] Cantero-Recasens G., Fandos C., Rubio-Moscardo F., Valverde M.A., Vicente R. (2010). The asthma-associated ORMDL3 gene product regulates endoplasmic reticulum-mediated calcium signaling and cellular stress. Hum. Mol. Genet..

[28] Himes B.E., Hunninghake G.M., Baurley J.W., Rafaels N.M., Sleiman P., Strachan D.P., Wilk J.B., Willis-Owen S.A., Klanderman B., Lasky-Su J. (2009). Genome-wide association analysis identifies PDE4D as an asthma-susceptibility gene. Am. J. Hum. Genet..

[29] Murphy A., Tantisira K.G., Soto-Quirós M.E., Avila L., Klanderman B.J., Lake S., Weiss S.T., Celedón J.C. (2009). PRKCA: a positional candidate gene for body mass index and asthma. Am. J. Hum. Genet..

[30] Sharma S., Raby B.A., Hunninghake G.M., Soto-Quirós M., Avila L., Murphy A.J., Lasky-Su J., Klanderman B.J., Sylvia J.S., Weiss S.T., Celedón J.C. (2009). Variants in TGFB1, dust mite exposure, and disease severity in children with asthma. Am. J. Respir. Crit. Care Med..

[31] Hunninghake G.M., Soto-Quirós M.E., Lasky-Su J., Avila L., Ly N.P., Liang C., Klanderman B.J., Raby B.A., Gold D.R., Weiss S.T., Celedón JC. (2008). Dust mite exposure modifies the effect of functional IL10 polymorphisms on allergy and asthma exacerbations. J. Allergy Clin. Immunol..

[32] DeMeo D.L., Lange C., Silverman E.K., Sentern J.M., Drazen J.M., Barth M.J., Laird N., Weiss S.T. (2002). Univariate and multivariate family-based association analysis of the IL-13 ARG130GLN polymorphism in the Childhood Asthma Management Program. Genet. Epidemiol..

[33] Raby B.A., Soto-Quiros M.E., Avila L., Lake S.L., Murphy A., Liang C., Fournier E., Spesny M., Sylvia J.S., Verner A., Hudson T.J., Klanderman B.J., Freimer N.B., Silverman E.K., Celedón J.C. (2007). Sex-specific linkage to total serum immunoglobulin E in families of children with asthma in Costa Rica. Hum. Mol. Genet..

[34] Imamura M., Okunishi K., Ohtsu H., Nakagome K., Harada H., Tanaka R., Yamamoto K., Dohi M. (2009). Pravastatin attenuates allergic airway inflammation by suppressing antigen sensitisation, interleukin 17 production and antigen presentation in the lung. Thorax.

[35] Saenz S.A., Siracusa M.C., Perrigoue J.G., Spencer S.P., Urban J.F., Tocker J.E., Budelsky A.L., Kleinschek M.A., Kastelein R.A., Kambayashi T., Bhandoola A., Artis D. (2010). IL25 elicits a multipotent progenitor cell population that promotes T(H)2 cytokine responses. Nature.

[36] Gusareva E.S., Bragina E.J., Buinova S.N., Chernyak B.A., Chernyak B.A., Puzyrev V.P., Ogorodova L.M., Lipoldová M. (2009). Chromosome 12q24.3 controls sensitization to cat allergen in patients with asthma from Siberia, Russia. Immunol. Lett..

[37] Gudbjartsson D.F., Bjornsdottir U.S., Halapi E., Helgadottir A., Helgadottir A., Sulem P., Jonsdottir G.M., Thorleifsson G., Helgadottir H., Steinthorsdottir V. (2009). Sequence variants affecting eosinophil numbers associate with asthma and myocardial infarction. Nat. Genet..

[38] Celedón J.C., Soto-Quiros M.E., Avila L., Lake S.L., Liang C., Fournier E., Spesny M., Hersh C.P., Sylvia J.S., Hudson T.J. (2007). Significant linkage to airway responsiveness on chromosome 12q24 in families of children with asthma in Costa Rica. Hum. Genet..

[39] Raby B.A., Silverman E.K., Lazarus R., Lange C., Kwiatkowski D.J., Weiss S.T. (2003). Chromosome 12q harbors multiple genetic loci related to asthma and asthma-related phenotypes. Hum. Mol. Genet..

[40] Randolph A.G., Lange C., Silverman E.K., Lazarus R., Silverman E.S., Raby B., Brown A., Ozonoff A., Richter B., Weiss S.T. (2004). The IL12B gene is associated with asthma. Am. J. Hum. Genet..

[41] Silverman E.K., Kwiatkowski D.J., Sylvia J.S., Lazarus R., Drazen J.M., Lange C., Laird N.M., Weiss S.T. (2003). Family-based association analysis of beta2-adrenergic receptor polymorphisms in the childhood asthma management program. J. Allergy Clin. Immunol..

[42] Lin M.T., Simon D.K., Ahn C.H., Kim L.M., Beal M.F. (2002). High aggregate burden of somatic mtDNA point mutations in aging and Alzheimer's disease brain. Hum. Mol. Genet..

[43] Simon D.K., Lin M.T., Zheng L., Liu G.J., Liu G.J., Ahn C.H., Kim L.M., Mauck W.M., Twu F., Beal M.F., Johns D.R. (2004). Somatic mitochondrial DNA mutations in cortex and substantia nigra in aging and Parkinson's disease. Neurobiol. Aging.

[44] Reeve A.K., Krishnan K.J., Turnbull D. (2008). Mitochondrial DNA mutations in disease, aging, and neurodegeneration. Ann. NY Acad. Sci..

[45] Reddy P.H. (2008). Mitochondrial medicine for aging and neurodegenerative diseases. Neuromol. Med..

[46] Tońska K., Sołyga A., Bartnik E. (2009). Mitochondria and aging: innocent bystanders or guilty parties?. J. Appl. Genet..

[47] Beal M.F. (2005). Mitochondria take center stage in aging and neurodegeneration. Ann. Neurol..

[48] Lin M.T., Beal M.F. (2006). Mitochondrial dysfunction and oxidative stress in neurodegenerative diseases. Nature.

[49] Cooper J.M., Mann V.M., Schapira A.H. (1992). Analyses of mitochondrial respiratory chain function and mitochondrial DNA deletion in human skeletal muscle: Effect of ageing. J. Neurol. Sci..

[50] Corral-Debrinski M., Horton T., Lott M.T., Shoffner J.M., Beal M.F., Wallace D.C. (1992). Mitochondrial DNA deletions in human brain: regional variability and increase with advanced age. Nat. Genet..

[51] Cortopassi G.A., Shibata D., Soong N.W., Arnheim N. (1992). A pattern of accumulation of a somatic deletion of mitochondrial DNA in aging human tissues. Proc. Natl. Acad. Sci. USA.

[52] Lee H.C., Pang C.Y., Hsu H.S., Wei Y.H. (1994). Differential accumulations of 4,977 bp deletion in mitochondrial DNA of various tissues in human ageing. Biochim. Biophys. Acta.

[53] Schwarze S.R., Lee C.M., Chung S.S., Roecker E.B., Weindruch R., Aiken J.M. (1995). High levels of mitochondrial DNA deletions in skeletal muscle of old rhesus monkeys. Mech. Ageing Dev..

[54] Lim P., Cheng Y., Wei Y. (2000). Large-scale mitochondrial DNA deletions in skeletal muscle of patients with end-stage renal disease. Free Rad. Biol. Med..

[55] Jessie B.C., Sun C.Q., Wallace D.C., Petros J.A. (2001). Accumulation of mitochondrial DNA deletions in the malignant prostate of patients of different ages. Exp. Gerontol..

[56] Hirai K., Aliev G., Nunomura A., Nunomura A., Fujioka H., Russell R.L., Atwood C.S., Johnson A.B., Kress Y., Vinters H.V. (2001). Mitochondrial abnormalities in Alzheimer's disease. J. Neurosci..

[57] Swerdlow R.H. (2007). Treating neurodegeneration by modifying mitochondria: potential solutions to a “complex” problem. Antioxid. Redox Signal..

[58] DiMauro S., Schon E.A. (2008). Mitochondrial disorders in the nervous system. Annu. Rev. Neurosci..

[59] Reddy P.H. (2007). Mitochondrial dysfunction in aging and Alzheimer's disease: strategies to protect neurons. Antioxid. Redox Signal..

[60] Reddy P.H., Beal M.F. (2005). Are mitochondria critical in the pathogenesis of Alzheimer's disease?. Brain Res. Brain Res. Rev..

[61] Anderson S., Bankier A.T., Barrell B.G., de Bruijn M.H., Coulson A.R., Drouin J., Eperon I.C., Nierlich D.P., Roe B.A., Sanger F., Schreier P.H., Smith A.J., Staden R., Young I.G. (1981). Sequence and organization of the human mitochondrial genome. Nature.

[62] Bucchieri F., Puddicombe S.M., Lordan J.L., Richter A., Konstantinidis A., Holloway J.W., Thornber M., Puddicombe S.M., Buchanan D., Wilson S.J., Djukanović R., Holgate S.T., Davies D.E. (2002). Asthmatic bronchial epithelium is more susceptible to oxidant-induced apoptosis. Am. J. Respir. Cell Mol. Biol..

[63] Truong-Tran A.Q., Grosser D., Ruffin R.E., Murgia C., Zalowski P.D. (2003). Apoptosis in the normal and inflamed airway epithelium: role of zinc in epithelial protection and procaspase-3 regulation. Biochem. Pharmacol..

[64] Reddy P.H. (2006). Mitochondrial oxidative damage in aging and Alzheimer's disease: implications for mitochondrially targeted antioxidant therapeutics. J. Biomed. Biotechnol..

[65] Reddy P.H. (2006). Amyloid precursor protein-mediated free radicals and oxidative damage: implications for the development and progression of Alzheimer's disease. J. Neurochem..

[66] Vignola A.M., Scichilone N., Bousquet J., Bonsignore G., Bellia V. (2003). Aging and asthma: pathophysiological mechanisms. Allergy.

[67] Braman S.S. (1993). Asthma in the elderly patient. Clin. Chest Med..

[68] Isoaho R., Puolijoki H., Huhti E., Kivelä S.L., Tala E. (1994). Prevalence of asthma in elderly Finns. J. Clin. Epidemiol..

[69] Dyer C.A., Hill S.L., Stockley R.A., Sinclair A.J. (1999). Quality of life in elderly subjects with a diagnostic label of asthma from general practice registers. Eur. Respir. J..

[70] Dow L., Fowler L., Phelps L., Waters K., Coggon D., Kinmonth A.L., Holgate S.T. (2001). Prevalence of untreated asthma in a population sample of 6000 older adults in Bristol, UK. Thorax.

[71] Thurlbeck W.M. (1991). Morphology of Aging Lung.

[72] Yung R.L. (2000). Changes in immune function with age. Rheum. Dis. Clin. North Am..

[73] Servais S., Boussouar A., Molnar A., Douki T., Pequignot J.M., Favier R. (2005). Age-related sensitivity to lung oxidative stress during ozone exposure. Free Radic. Res..

[74] Fahn H.J., Wang L.S., Kao S.H., Chang S.C., Huang M.H., Wei Y.H. (1998). Smoking-associated mitochondrial DNA mutations and lipid peroxidation in human lung tissues. Am. J. Respir. Cell Mol. Biol..

[75] Wood L.G., Gibson P.G., Garg M.L. (2003). Biomarkers of lipid peroxidation, airway inflammation and asthma. Eur. Respir. J..

[76] Hayashi T., Ishii A., Nakai S., Hasegawa K. (2004). Ultrastructure of goblet-cell metaplasia from Clara cell in the allergic asthmatic airway inflammation in a mouse model of asthma *in vivo*. Virchows Arch.

[77] Konrádová V., Copová C., Suková B., Houstĕk J. (1985). Ultrastructure of the bronchial epithelium in three children with asthma. Pediatr. Pulmonol..

[78] Comhair S.A., Bhathena P.R., Dweik R.A., Kavuru M., Erzurum S.C. (2000). Rapid loss of superoxide dismutase activity during antigen-induced asthmatic response. Lancet.

[79] Comhair S.A., Ricci K.S., Arroliga M., Lara A.R., Dweik R.A., Song W., Hazen S.L., Bleecker E.R., Busse W.W., Chung K.F. (2005). Correlation of systemic superoxide dismutase deficiency to airflow obstruction in asthma. Am. J. Respir. Crit. Care Med..

[80] De Raeve H.R., Thunnissen F.B., Kaneko F.T., Guo F.H., Lewis M., Kavuru M.S., Secic M., Thomassen M.J., Erzurum S.C. (1997). Decreased Cu, Zn-SOD activity in asthmatic airway epithelium: correction by inhaled corticosteroid *in vivo*. Am. J. Physiol..

[81] Wu W., Samoszuk M.K., Comhair S.A., Thomassen M.J., Thomassen M.J., Farver C.F., Dweik R.A., Kavuru M.S., Erzurum S.C., Hazen S.L. (2000). Eosinophils generate brominating oxidants in allergen-induced asthma. J. Clin. Invest..

[82] Thomassen M.J., Raychaudhuri B., Dweik R.A., Farver C., Buhrow L., Malur A., Connors M.J., Drazba J., Hammel J., Erzurum S.C., Kavuru M.S. (1999). Nitric oxide regulation of asthmatic airway inflammation with segmental allergen challenge. J. Allergy Clin. Immunol..

[83] Fitzpatrick A.M., Teague W.G., Holguin F, Yeh M., Brown L.A. (2009). Severe asthma research program. Airway glutathione homeostasis is altered in children with severe asthma: evidence for oxidant stress. J. Allergy Clin. Immunol..

[84] Comhair S.A., Bhathena P.R., Farver C., Thunnissen F.B., Erzurum S.C. (2001). Extracellular glutathione peroxidase induction in asthmatic lungs: evidence for redox regulation of expression in human airway epithelial cells. FASEB J..

[85] MacPherson J.C., Comhair S.A., Erzurum S.C., Klein D.F., Lipscomb M.F., Kavuru M.S., Samoszuk M.K., Hazen S.L. (2001). Eosinophils are a major source of nitric oxide-derived oxidants in severe asthma: characterization of pathways available to eosinophils for generating reactive nitrogen species. J. Immunol..

[86] Takeda K., Gelfand E.W. (2009). Mouse models of allergic diseases. Curr. Opin. Immunol..

[87] Nials A.T., Uddin S. (2008). Mouse models of allergic asthma: acute and chronic allergen challenge. Dis. Model. Mech..

[88] Finkelman F.D., Wills-Karp M. (2008). Usefulness and optimization of mouse models of allergic airway disease. J. Allergy Clin. Immunol..

[89] Wenzel S., Holgate S.T. (2006). The mouse trap: It still yields few answers in asthma. Am. J. Respir. Crit. Care Med..

[90] Park C.S., Kim T.B., Lee K.Y., Moon K.A., Bae Y.J., Jang M.K., Cho Y.S., Moon H.B. (2009). Increased oxidative stress in the airway and development of allergic inflammation in a mouse model of asthma. Ann. Allergy Asthma Immunol..

[91] Konga D.B., Kim Y., Hong S.C., Roh Y.M., Lee C.M., Kim K.Y., Lee S.M. (2009). Oxidative stress and antioxidant defenses in asthmatic murine model exposed to printer emissions and environmental tobacco smoke. J. Environ. Pathol. Toxicol. Oncol..

[92] Bharadwaj A.S., Agrawal D.K. (2007). Flt3 ligand generates morphologically distinct semimature dendritic cells in ovalbumin-sensitized mice. Mol. Pathol..

[93] Zhang L., Wang M., Kang X., Boontheung P., Li N., Nel A.E., Loo J.A. (2009). Oxidative stress and asthma: proteome analysis of chitinase-like proteins and FIZZ1 in lung tissue and bronchoalveolar lavage fluid. J. Proteome Res..

[94] Inoue K., Takano H., Koike E., Warabi E., Yanagawa T., Yanagisawa R., Ishii T. (2009). Peroxiredoxin I is a negative regulator of Th2-dominant allergic asthma. Int. Immunopharmacol..

[95] Lee K.S., Park H.S., Park S.J., Kim S.R., Min K.H., Jin S.M., Li L., Lee Y.C. (2006). An antioxidant modulates expression of receptor activator of NF-kappaB in asthma. Exp. Mol. Med..

[96] Lee K.S., Kim S.R., Park S.J., Min K.H., Lee H.K., Park H.S., Min K.H., Jin S.M., Lee Y.C. (2006). Antioxidant down-regulates interleukin-18 expression in asthma. Mol. Pharmacol..

[97] Castro S.M., Guerrero-Plata A., Suarez-Real G., Adegboyega P.A., Colasurdo G.N., Khan A.M., Garofalo R.P., Casola A. (2006). Antioxidant treatment ameliorates respiratory syncytial virus-induced disease and lung inflammation. Am. J. Respir. Crit. Care Med..

[98] Mabalirajan U., Aich J., Leishangthem G.D., Sharma S.K., Dinda A.K., Ghosh B. (2009). Effects of vitamin E on mitochondrial dysfunction and asthma features in an experimental allergic murine model. J. Appl. Physiol..

[99] Mabalirajan U., Dinda A.K., Sharma S.K., Ghosh B. (2009). Esculetin restores mitochondrial dysfunction and reduces allergic asthma features in experimental murine model. J. Immunol..

[100] Lee M., Kim S., Kwon O.K., Oh S.R., Lee H.K., Ahn K. (2009). Anti-inflammatory and anti-asthmatic effects of resveratrol, a polyphenolic stilbene, in a mouse model of allergic asthma. Int. Immunopharmacol..

[101] Dittrich A.M., Meyer H.A., Krokowski M., Quarcoo D., Ahrens B., Kube S.M., Witzenrath M., Esworthy R.S., Chu F.F., Hamelmann E. (2009). Glutathione peroxidase-2 protects from allergen-induced airway inflammation in mice. Eur. Respir. J..

[102] Chang H.H., Chen C.S., Lin J.Y. (2009). High dose vitamin C supplementation increases the Th1/Th2 cytokine secretion ratio, but decreases eosinophilic infiltration in bronchoalveolar lavage fluid of ovalbumin-sensitized and challenged mice. J. Agric. Food Chem..

[103] Ahmad T., Mabalirajan U., Ghosh B., Agrawal A. (2010). Altered asymmetric dimethyl arginine metabolism in allergically inflamed mouse lungs. Am. J. Respir. Cell Mol. Biol..

[104] Okamoto N., Murata T., Tamai H., Tanaka H., Nagai H. (2006). Effects of alpha tocopherol and probucol supplements on allergen-induced airway inflammation and hyperresponsiveness in a mouse model of allergic asthma. Int. Arch. Allergy Immunol..

[105] Mehta A.K., Arora N., Gaur S.N., Singh B.P. (2009). Choline supplementation reduces oxidative stress in mouse model of allergic airway disease. Eur. J. Clin. Invest..

[106] Braskett M., Riedl M.A. (2010). Novel antioxidant approaches to the treatment of upper airway inflammation. Curr. Opin. Allergy Clin. Immunol..

[107] Gazdík F., Gvozdjáková A., Nádvorníková R., Repická L, Repická L., Jahnová E., Kucharská J., Piják M.R., Gazdíková K. (2002). Decreased levels of coenzyme Q(10) in patients with bronchial asthma. Allergy.

[108] Gazdik F., Gvozdjakova A., Horvathova M., Weissova S., Kucharska J., Pijak M.R., Gazdikova K. (2002). Levels of coenzyme Q10 in asthmatics. Bratisl. Lek. Listy.

[109] Gvozdjáková A., Kucharská J., Bartkovjaková M., Gazdíková K., Gazdík F.E. (2005). Coenzyme Q10 supplementation reduces corticosteroids dosage in patients with bronchial asthma. Biofactors.

[110] Domenighetti G., Suter P.M., Schaller M.D., Ritz R., Perret C. (1997). Treatment with *N*-acetylcysteine during acute respiratory distress syndrome: a randomized, double-blind, placebo-controlled clinical study. J. Crit. Care.

[111] Konrad F., Schoenberg M.H., Wiedmann H., Kilian J., Georgieff M. (1995). The application of *n*-acetylcysteine as an antioxidant and mucolytic in mechanical ventilation in intensive care patients. A prospective, randomized, placebo-controlled, double-blind study. Anaesthesist.

[112] Suter P.M., Domenighetti G., Schaller M.D., Laverrière M.C., Ritz R., Perret C. (1994). *N*-acetylcysteine enhances recovery from acute lung injury in man. A randomized, double-blind, placebo-controlled clinical study. Chest.

[113] Moradi M., Mojtahedzadeh M., Mandegari A., Soltan-Sharifi M.S., Najafi A., Khajavi M.R., Hajibabayee M., Ghahremani M.H. (2009). The role of glutathione-S-transferase polymorphisms on clinical outcome of ALI/ARDS patient treated with *N*-acetylcysteine. Respir. Med..

[114] Devereux G., Turner S.W., Craig L.C., McNeill G., Martindale S., Harbour P.J., Helms P.J., Seaton A. (2006). Low maternal vitamin E intake during pregnancy is associated with asthma in 5-year-old children. Am. J. Respir. Crit. Care Med..

[115] Pearson P.J., Lewis S.A., Britton J., Fogarty A. (2004). Vitamin E supplements in asthma: a parallel group randomised placebo controlled trial. Thorax.

[116] Romieu I., Sienra-Monge J.J., Ramírez-Aguilar M., Moreno-Macías H., Reyes-Ruiz N.I., Estela del Río-Navarro B., Hernández-Avila M., London S.J. (2004). Genetic polymorphism of GSTM1 and antioxidant supplementation influence lung function in relation to ozone exposure in asthmatic children in Mexico City. Thorax.

[117] Shahar E., Hassoun G., Pollack S. (2004). Effect of vitamin E supplementation on the regular treatment of seasonal allergic rhinitis. Ann. Allergy Asthma Immunol..

[118] Sienra-Monge J.J., Ramirez-Aguilar M., Moreno-Macias H., Reyes-Ruiz N.I., Del Río-Navarro B.E., Ruiz-Navarro M.X., Hatch G., Crissman K., Slade R., Devlin R.B., Romieu I. (2004). Antioxidant supplementation and nasal inflammatory responses among young asthmatics exposed to high levels of ozone. Clin. Exp. Immunol..

[119] Papas K.A., Sontag M.K., Pardee C., Sokol RJ., Sagel S.D., Accurso F.J., Wagener J.S. (2008). A pilot study on the safety and efficacy of a novel antioxidant rich formulation in patients with cystic fibrosis. J. Cyst. Fibros..

[120] Szeto H.H. (2008). Development of mitochondria-targeted aromatic-cationic peptides for neurodegenerative diseases. Ann. NY Acad. Sci..

[121] Murphy M.P., Smith R.A. (2007). Targeting antioxidants to mitochondria by conjugation to lipophilic cations. Annu. Rev. Pharmacol. Toxicol..

[122] Szeto H.H. (2006). Cell-permeable, mitochondrial-targeted, peptide antioxidants. AAPS J..

[123] Szeto H.H. (2006). Mitochondria-targeted peptide antioxidants: Novel neuroprotective agents. AAPS J..

[124] Petri S., Kiaei M., Damiano M., Hiller A., Wille E., Manfredi G., Calingasan N.Y., Szeto H.H., Beal M.F. (2006). Cell-permeable peptide antioxidants as a novel therapeutic approach in a mouse model of amyotrophic lateral sclerosis. J. Neurochem..

[125] Yang L., Zhao K., Calingasan N.Y., Luo G., Szeto H.H., Beal M.F. (2009). Mitochondria targeted peptides protect against 1-methyl-4-phenyl-1,2,3,6-tetrahydropyridine neurotoxicity. Antioxid. Redox Signal..

[126] Reddy T.P., Manczak M., Calkins M.J., Mao P., Reddy A.P., Shirendeb U., Park B., Reddy P.H. (2011). Toxicity of neurons treated with herbicides and neuroprotection by mitochondria-targeted antioxidant SS31. Int. J. Environ. Res. Public Health.

[127] Manczak M., Mao P., Calkins M.J., Cornea A., Reddy A.P., Murphy M.P., Szeto H.H., Park B., Reddy P.H. (2010). Mitochondria-targeted antioxidants protect against amyloid-beta toxicity in Alzheimer's disease neurons. J. Alzheimers Dis..

